# Association of culprit lesion plaque characteristics with flow restoration post-fibrinolysis in ST-segment elevation myocardial infarction: an intravascular ultrasound-virtual histology study

**DOI:** 10.1186/s43044-020-00121-w

**Published:** 2020-12-09

**Authors:** Raghavendra Rao K, Sreenivas Reddy, Jeet Ram Kashyap, Vadivelu Ramalingam, Debabrata Dash, Vikas Kadiyala, Suraj Kumar, Hithesh Reddy, Jaspreet Kaur, Ashok Kumar, Naindeep Kaur, Anish Gupta

**Affiliations:** 1 Department of Cardiology, Government Medical College and Hospital, Sector 32, Chandigarh, 160030 India; 2 Department of Neurology, Post Graduate Institute of Medical Education and Research (PGIMER), Chandigarh, 160012 India

**Keywords:** ST-segment elevation myocardial infarction, Fibrinolysis, Intravascular ultrasound, Virtual histology-intravascular ultrasound, Necrotic core, TIMI flow

## Abstract

**Background:**

Not every patient achieves normal coronary flow following fibrinolysis in STEMI (ST-segment elevation myocardial infarction). The culprit lesion plaque characteristics play a prominent role in the coronary flow before and during percutaneous coronary intervention. The main purpose was to determine the culprit lesion plaque features by virtual histology-intravascular ultrasound (VH-IVUS) in patients with STEMI following fibrinolysis in relation to baseline coronary angiogram TIMI (thrombolysis in myocardial infarction) flow. Pre-intervention IVUS was undertaken in 61 patients with STEMI after successful fibrinolysis. After the coronary angiogram, they were separated into the TIMI1–2 flow group (*n* = 31) and TIMI 3 flow group (*n* = 30). Culprit lesion plaque composition was evaluated by VH-IVUS.

**Results:**

On gray-scale IVUS, the lesion external elastic membrane cross-sectional area (EEM CSA) was significantly higher in the TIMI 1–2 groups as compared to the TIMI 3 group (15.71 ± 3.73 mm^2^ vs 13.91 ± 2.94 mm^2^, *p* = 0.041) with no significant difference in plaque burden (82.42% vs. 81.65%, *p* = 0.306) and plaque volume (108.3 mm^3^ vs. 94.3 mm^3^, *p* = 0.194). On VH-IVUS, at the minimal luminal area site (MLS), the fibrous area (5.83 mm^2^ vs. 4.37 mm^2^, *p* = 0.024), necrotic core (NC) area (0.95 mm^2^ vs. 0.59 mm^2^, *p* < 0.001), and NC percentage (11% vs. 7.1%, *p* = 0.024) were higher in the TIMI 1–2 groups in contrast to the TIMI 3 group. The absolute necrotic core (NC) volume (8.3 mm^3^ vs. 3.65 mm^3^, *p* < 0.001) and NC percentage (9.3% vs. 6.0%, *p* = 0.007) were significantly higher in the TIMI 1–2 groups as compared to the TIMI 3 group. Absolute dense calcium (DC) volume was higher in TIMI 1–2 groups with a trend towards significance (1.0 mm^3^ vs.0.75 mm^3^, *p* = 0.051). In multivariate analysis, absolute NC volume was the only independent predictor of TIMI 1–2 flow (odds ratio = 1.561; 95% CI 1.202–2.026, *p* = 0.001). Receiver operating characteristic curves showed absolute NC volume has best diagnostic accuracy (AUC = 0.816, *p* < 0.001) to predict TIMI 1–2 flow with an optimal cutoff value of 4.5 mm^3^ with sensitivity and specificity of 79% and 61%, respectively.

**Conclusions:**

This study exemplifies that the necrotic core component of the culprit lesion plaque in STEMI is associated with the coronary flow after fibrinolysis. The absolute necrotic core volume is a key determinant of flow restoration post-fibrinolysis and aids in prognostication of less than TIMI 3 flow.

## Background

Cardiovascular disease (CVD) afflicts the entire world and is the prominent cause of mortality in developing countries accounting for 80% of all cardiovascular-related deaths [1]. ST-segment elevation myocardial infarction (STEMI) is associated with significant morbidity and mortality. Timely and adequate reperfusion has been undoubtedly proven to be beneficial. Primary percutaneous coronary intervention (PPCI) achieves reperfusion with few complications as compared to thrombolysis and is considered the standard therapy [2–4]. However, as compared to the developed countries, the developing countries face a huge gap in the STEMI care due to limited health care infrastructure, financial constraints, poor accessibility, or non-availability, making fibrinolysis a reasonable alternative to PPCI [5–8]. The finding of less than TIMI 3 flow in the culprit vessel after fibrinolysis is in consonance with higher rates of complications such as recurrent ischemia, heart failure, and diminished salvage of myocardium which are linked to increased mortality [9], whereas the existence of TIMI 3 flow prior to primary PCI is known to improve early and late survival with favorable long-term outcomes [10, 11]. Even after fibrinolysis, a sizeable proportion of patients fails to achieve TIMI 3 flow on coronary angiography [12].

Intravascular ultrasound (IVUS) is a pivotal tool for the quantitative and qualitative assessment of the coronary atherosclerosis. Virtual histology IVUS (VH-IVUS) provides additional quantitative information on the plaque composition and characterizes atherosclerotic plaque phenotypes with an excellent correlation with histopathological examination with high predictive accuracies varying from 87.1 to 96.5% [13, 14].

## Aim of the work

The purpose of the study was to demonstrate the culprit lesion plaque traits in subjects with STEMI following fibrinolysis in relation to TIMI flow on coronary angiogram.

a) Comparison of culprit lesion plaque composition by VH-IVUS in patients with TIMI 3 versus TIMI 1–2 flow in STEMI patients post fibrinolysis.

b) Identifying VH-IVUS predictors of TIMI 1–2 flow.

## Methods

### Study population

Between June 2017 and November 2019, a total of 495 patients with the acute coronary syndrome (ACS) to the tertiary care center were screened as part of IVUS in ACS study. A total of 342 patients were diagnosed with STEMI, of which 61 patients underwent successful fibrinolysis (ST-segment resolution by ≥ 50% from baseline elevation within 90 min and remission of clinical symptoms) and referred for coronary angiogram were included. ST-segment elevation myocardial infarction (STEMI) was determined by continuous chest pain lasting > 30 min, a new ST-segment elevation ≥ 2 mm on at least 2 contiguous electrocardiogram leads and with a rise in troponins or creatine kinase-myocardial band (CK-MB) > 3 times normal value. The identification of the culprit vessel and lesion was based on electrocardiographic changes, echocardiogram findings, and angiographic lesion morphology.

Patients with unstable angina, non-ST-segment elevation myocardial infarction (NSTEMI), severe renal dysfunction (creatinine clearance < 30 ml/minute), coronary vessels not feasible for IVUS imaging, unstable hemodynamics, Killip class III/IV, prior history of angioplasty or coronary bypass surgery, and unwilling to give consent were excluded. A written informed consent was obtained from all the patients prior to the study initiation and was approved by the Institutional Ethics Committee. All patients underwent peripheral blood examination for hemogram, renal function tests, total cholesterol along with fractions, and CK-MB levels. All the procedures were performed in conformity with the Good Clinical Practice (GCP) principles as mentioned in the Declaration of Helsinki.

### Interventional procedures

The coronary angiogram and percutaneous coronary intervention were performed either from radial or femoral routes after preloading with aspirin, clopidogrel or prasugrel or ticagrelor in standard doses. Unfractionated heparin was administered intravenously at a dose of 70–100 U/kg and titration made to achieve targeted activated clotting time 250–300 s during the PCI procedure. IVUS imaging was undertaken immediately after the coronary angiogram following the administration of 200–300 mcg of intracoronary nitroglycerine using a 20-MHz, 2.9 French, Eagle Eye® Platinum RX digital IVUS catheter (Eagle Eye, Philips Volcano, San Diego, CA, USA). IVUS catheter advanced 15 mm distal to the lesion and pull back taken till the aorto-ostial junction with a motorized automatic pullback system (R-100 pull back, Volcano) at a constant speed of 0.5 mm/s before any balloon pre-dilatation.

### Angiographic analysis

A cine frame rate of 15/s was adopted for performing coronary angiography and PCI. A computerized software Medis Q Angio® XA 7.3 (Medis Medical Imaging Systems, Leiden, the Netherlands) was utilized for analysis of the baseline coronary angiograms by two independent observers (VK and JRK) blinded to patient clinical data. After analyzing the baseline coronary angiograms, two groups constituted the TIMI 3 flow group and TIMI 1–2 flow groups. For the objective assessment of coronary flow, corrected thrombolysis in myocardial infarction frame count (CTFC) was utilized and cine frame count calculated by multiplication of 30 and divided by 15 to be reported as standard methods [15]. Thrombus grading from 0 to 5 as described by Gibson et al. [16] and the coronary collaterals grading by Rentrop et al. were estimated [17].

### Gray-scale and virtual histology-IVUS analysis

The IVUS images obtained were recorded in digital media and archived in a DVD-ROM for offline analysis. Independent observers unbeknownst of patient characteristics or angiograms (HR and SK) undertook the analysis. The IVUS measurements and analysis were conducted in congruence with current standard methods [18]. An Echoplaque 4.3.12J computerized software (Indec Medical Systems, Inc., Santa Clara, CA, USA) was used for the analysis. The culprit lesion considered was the smallest lumen site. The image slices situated within 10 mm on either side of the lesion were the proximal and distal reference sites with the least plaque burden and no major side branch. The software automatically detects the lumen and media-adventitia interface. Manual correction if required to be confirmed and the calculated results are displayed. External elastic membrane (EEM) and lumen cross-sectional areas (CSA) were measured. Plaque and media (P&M) CSA was calculated as EEM minus lumen CSA. Plaque burden calculation was made as plaque and media CSA/EEM CSA multiplied by 100. The lesion was considered as the image slice site with the smallest lumen CSA along with the largest EEM and P&M CSA. The ratio between lesion site EEM CSA and mean of the proximal and distal reference EEM CSA was the remodeling index. Positive remodeling and negative remodeling were remodeling index > 1.05 and < 0.95, respectively [19]. A cross-sectional analysis was carried out at the minimal lumen area site. Volumetric analysis was executed over a 10-mm vascular segment with minimal luminal area site considered as center and calculations made by Simpson’s rule. Virtual histology analysis images exhibit four major tissue components to be displayed: fibrous as green, fibrofatty as yellow-green, dense calcium as white, and necrotic core as red. The measurements were expressed in terms of percentage of plaque area/volumes or as absolute units. The TCFA (thin-cap fibroatheroma) was contemplated when a lesion fulfilled the said criteria in at least 3 consecutive images slices (a) confluent necrotic core ≥ 10% of plaque area in direct contact with the lumen (b) subtending an arc > 30° necrotic core (c) plaque burden of ≥ 40% [20].

### Assessment of reproducibility

Intraobserver variability was assessed by analyzing a set of IVUS pullbacks twice by the same person at an interval of 3 months. The corresponding intra-class correlation coefficient (ICC) for repeated measurement was 0.81 (95% confidence interval 0.68–0.89) for lumen measurements and 0.91 (95% confidence interval 0.85–0.94) for volumes. The intraclass correlation coefficient for interobserver variability was 0.87 (95% confidence interval 0.74–0.93) for lumen measurements and 0.94 (95% confidence interval 0.88–0.97) for volume, suggestive of acceptable concordance.

### Statistical analysis

A SPSS version 23.0 (SPSS, Inc., Chicago, Illinois) utilized for statistical analysis. Categorical data was presented as percentages (%) and frequencies. Continuous variables were evaluated using Shapiro-Wilk test and reported as mean with standard deviation when distributed normally and median with the 25th and 75th percentiles if skewed distribution. Bivariate analysis was done to determine associations of categorical variables within the two study groups using chi-squared test/Fisher’s exact test as appropriate, and for continuous variables, we used independent *t* test/Mann-Whitney *U* test. Variables that were significant (*p* < 0.05) in the bivariate analysis were considered for multivariate analysis. We performed backward stepwise logistic regression with entry *p* value as 0.20 and exit *p* value as 0.05. The model performance was judged by Cox and Snell *R*^2^ and classification accuracy. A *p* value < 0.05 was considered for statistical significance for the analyses.

## Results

### Patient clinical characteristics

The study comprised 61 patients for analysis. All patients received fibrinolytic therapy, and on coronary angiogram, TIMI 3 flow was seen in 30 patients (49.1%), TIMI 2 was noted in 25 (40.9%), and TIMI 1 was observed in 6 (9.8%). The patient clinical characteristics at baseline are listed in Table 1. No difference was observed in age, hypertension, diabetes mellitus, smoking, and family history of premature coronary artery disease among the groups. Likewise, no significance was discerned in fibrinolytic agents use, symptom onset to fibrinolysis time, fibrinolysis to PCI time, and glycoprotein IIb/IIIa inhibitors use between the groups. Blood parameters such as hemoglobin, creatinine, lipids, and creatine kinase-MB were comparable among the groups. Left ventricular ejection fraction on echocardiogram was lower in the TIMI 1–2 groups in comparison to the TIMI 3 group (40.25 ± 6.34% vs 43.40 ± 4.28 %; *p* = 0.027).

**Table 1:** Baseline characteristics of the patients (*n* = 61)

Variables	TIMI flow 3 (*n* = 30)	TIMI flow 1–2 (*n* = 31)	*p* value
Age (years)	54.63 ± 8.98	57.10 ± 14.13	0.419
Male, *n* (%)	26 (86.7%)	26 (83.9%)	1.000
Hypertension, *n* (%)	5 (16.7%)	11 (35.5%)	0.095
Diabetes mellitus, *n* (%)	6 (20%)	7 (22.6%)	0.806
Smoking, *n* (%)	17 (56.7%)	12 (38.7%)	0.160
Family history of CAD, *n* (%)	3 (10%)	3 (9.7%)	1.000
BMI (kg/m^2^)	25.4 ± 4.17	25.10 ± 3.04	0.753
BSA (m^2^)	1.74 ± 0.13	1.74 ± 0.12	0.891
Symptom to thrombolysis time (hours)	4.77 ± 1.60	4.67 ± 1.47	0.809
Thrombolysis to PCI time (hours)	16.03 ± 1.37	15.71 ± 1.40	0.377
Hemoglobin (g/dL)	13.19 ± 1.85	13.31 ± 2.06	0.822
Creatinine (mg/dL)	1.1 ± 0.17	1.07 ± 0.18	0.485
Total cholesterol (mg/dL)	152.1 ± 52.85	139 ± 30.16	0.262
Triglycerides (mg/dL)	134.1 ± 47.18	119.2 ± 40.82	0.208
LDL-cholesterol (mg/dL)	100.8 ± 54.47	86.6 ± 33.42	0.247
HDL-cholesterol (mg/dL)	38.18 ± 9.05	38.84 ± 11.74	0.814
CK-MB (IU/l)	40 (36.25–53)	37 (31 − 97.75)	0.852
Ejection fraction, (%)	43.40 ± 4.28	40.25 ± 6.34	0.027
Thrombolysis, *n* (%)			0.656
Streptokinase	24 (80%)	22 (71%)	
Reteplase	6 (20%)	8 (25.8%)	
Tenecteplase	0 (0%)	1 (3.2%)	
GP IIb-IIIa inhibitor use, *n* (%)	11 (36.7%)	10 (32.3%)	0.717
Medications at admission (%)			
Antiplatelets (%)	2 (6.6%)	3 (9.6%)	1.000
Statins (%)	3 (10%)	4 (12.9%)	1.000
Beta blockers (%)	2 (6.6%)	5 (16.1%)	0.425
ACE I/ARBs (%)	4 (13.3%)	5 (16.1%)	1.000
OHAs (%)	3 (10%)	5 (16.1%)	0.707
Insulin (%)	2 (6.6%)	1 (3.2%)	0.612

Data are presented as mean ± SD, median (interquartile range), or *n* (%)

*CAD* coronary artery disease, *BMI* body mass index, *BSA* body surface area, *PCI* percutaneous coronary intervention, *LDL* low-density lipoprotein, *HDL* high-density lipoprotein, *CK-MB* creatine kinase-myocardial band (IU/l), *ACE I* angiotensin-converting-enzyme inhibitors, *ARB* angiotensin receptor blockers, *OHA* oral hypoglycemic agents

### Coronary angiographic and intervention procedural features

Angiographic traits and findings are listed in Table 2. The corrected TIMI frame count (CTFC) was significantly higher in the TIMI 1–2 groups as compared to the TIMI 3 group at baseline coronary angiogram (50.58 vs. 34.0; *p* < 0.001). On coronary angiography, the number of diseased vessels, ACC/AHA lesion type, TIMI thrombus grading, and collateral flow grades were comparable among the groups. The culprit vessel was LAD in the majority of patients in the TIMI 1–2 group in contrast to the TIMI 3 group (90.3% vs. 43.3%; *p* < 0.001). Quantitative coronary angiography (QCA) analysis between the groups was comparable with no statistically significant difference.

**Table 2:** Angiographic characteristics and procedure findings (*n* = 61)

Variables	TIMI flow 3 (*n* = 30)	TIMI flow 1–2 (*n* = 31)	*p* value
Culprit vessel, *n* (%)			< 0.001
LAD	13 (43.3%)	28 (90.3%)	
LCX	4 (13.3%)	1 (3.2%)	
RCA	13 (43.3%)	2 (6.5%)	
Diseased vessels, *n* (%)			
SVD	17 (56.7%)	21 (67.7%)	0.372
DVD	11 (36.7%)	6 (19.4%)	0.132
TVD	2 (6.7%)	4 (12.9%)	0.671
ACC/AHA Lesion type, *n* (%)			0.350
Type A	14 (46.7%)	13 (41.9%)	
Type B_1_	9 (30%)	10 (32.3%)	
Type B_2_	3 (10%)	7 (22.6%)	
Type C	4 (13.3%)	1 (3.2%)	
Baseline TIMI flow grade, *n* (%)			< 0.001
1	0 (0%)	6 (19.4%)	
2	0 (0%)	25 (80.6%)	
3	30 (100%)	0 (0%)	
Collateral flow grade (Rentrop), *n* (%)			1.000
0	30 (100%)	30 (96.8%)	
1	0 (0%)	0 (0%)	
2	0 (0%)	1 (3.2%)	
3	0 (0%)	0 (0%)	
TIMI Thrombus grading, *n* (%)			0.662
0	27 (90%)	24 (77.4%)	
1	2 (6.7%)	5 (16.1%)	
2	0 (0%)	0 (0%)	
3	1 (3.3%)	1 (3.2%)	
4	0 (0%)	1 (3.2%)	
5	0 (0%)	0 (0%)	
CTFC (baseline angiogram)	34 (28.82–42)	50.58 (44.7–61.17)	< 0.001
Quantitative coronary angiography data			
Obstruction diameter (mm)	0.86 (0.73–1.19)	0.95 (0.73–1.31)	0.549
Reference diameter (mm)	2.55 ± 0.38	2.56 ± 0.55	0.967
Diameter stenosis (%)	62.20 ± 10.15	59.40 ± 15.25	0.404
Area stenosis (%)	85.62 (78.58–91.32)	86.51 (70.29–91.07)	0.399

Data are presented as mean ± SD, median (interquartile range), or *n* (%)

*LAD* left anterior descending coronary artery, *LCX* left circumflex coronary artery, *RCA* right coronary artery, *SVD* single-vessel disease, *DVD* double vessel disease, *TVD* triple vessel disease, *TIMI* thrombolysis in myocardial infarction, *CTFC* corrected thrombolysis in myocardial infarction frame count, *PCI* percutaneous coronary intervention

### Gray-scale and VH-IVUS findings

The gray-scale IVUS findings are shown in Table 3. The lesion length exhibited no significance between the groups. The estimated values in proximal and distal reference sites were no different in the groups except for the distal reference plaque burden which was higher in the TIMI 1 or 2 group (36.92% vs. 31.4%; *p* = 0.027). Similarly, the gray-scale IVUS measurements at the minimal luminal area site (MLS) were comparable except for the lesion EEM CSA being higher in TIMI 1–2 groups in comparison to the TIMI 3 group (lesion EEM CSA: 15.71 ± 3.73 mm^2^ vs. 13.91 ± 2.94 mm^2^, *p* = 0.041). The remodeling index revealed no statistical difference (1.21 vs. 1.22; *p* = 0.946).

**Table 3:** Gray-scale IVUS findings (*n* = 61)

Variables	TIMI flow 3 (*n* = 30)	TIMI flow 1–2 (*n* = 31)	*p* value
IVUS lesion length (mm)	28.95 (19.40–39.62)	25.20 (19.70–31.90)	0.299
Proximal reference			
Lumen CSA (mm^2^)	8.65 (7.68–9.89)	9.86 (6.86–11.50)	0.328
EEM CSA (mm^2^)	13.21 (12.03–15.76)	15.65 (11.68–18.31)	0.124
Plaque burden (%)	33.28 (27.37–38.11)	36.11 (28.37–38.74)	0.585
Distal reference			
Lumen CSA (mm^2^)	5.95 ± 1.93	5.36 ± 1.65	0.212
EEM CSA (mm^2^)	9.21 (6.13–11.49)	8.48 (6.43–11.56)	0.828
Plaque burden (%)	31.40 (26.93–37.27)	36.92 (33–39.91)	0.027
Average/Mean Lumen CSA (mm^2^)	7.32 (6.39–8.80)	7.30 (5.60–8.59)	0.725
Average/Mean EEM CSA (mm^2^)	10.95 (9.61–13.31)	12.36 (8.87–14.93)	0.213
Lesion measurements			
Lesion minimum luminal diameter (mm)	1.56 (1.5–1.63)	1.57 (1.51–1.68)	0.436
Lesion maximum luminal diameter (mm)	2.0 (1.82–2.22)	2.06 (1.82–2.25)	0.670
Lesion Lumen CSA (mm^2^)	2.43 (2.15–2.71)	2.45 (2.11–2.87)	0.363
Lesion EEM CSA (mm^2^)	13.91 ± 2.94	15.71 ± 3.73	0.041
Lesion (P + M) CSA (mm^2^)	11.45 ± 2.85	13.13 ± 3.66	0.050
Lesion lumen area stenosis (%)	67.30 (60.39–71.18)	66.14 (55.12–71.57)	0.658
Lesion plaque burden (%)	81.65 (79.98–84.01)	82.42 (80.68–85.77)	0.306
Plaque volume (mm^3^)	94.3 (72.22–120.22)	108.3 (94.10–120.20)	0.194
Remodeling index	1.21 (1.06–1.42)	1.22 (1.03–1.50)	0.946

Data are presented as mean ± SD, median (interquartile range), or *n* (%)

*IVUS* intravascular ultrasound, *EEM CSA* external elastic membrane cross-sectional area, *P + M* plaque plus media

**Fig. 1:**
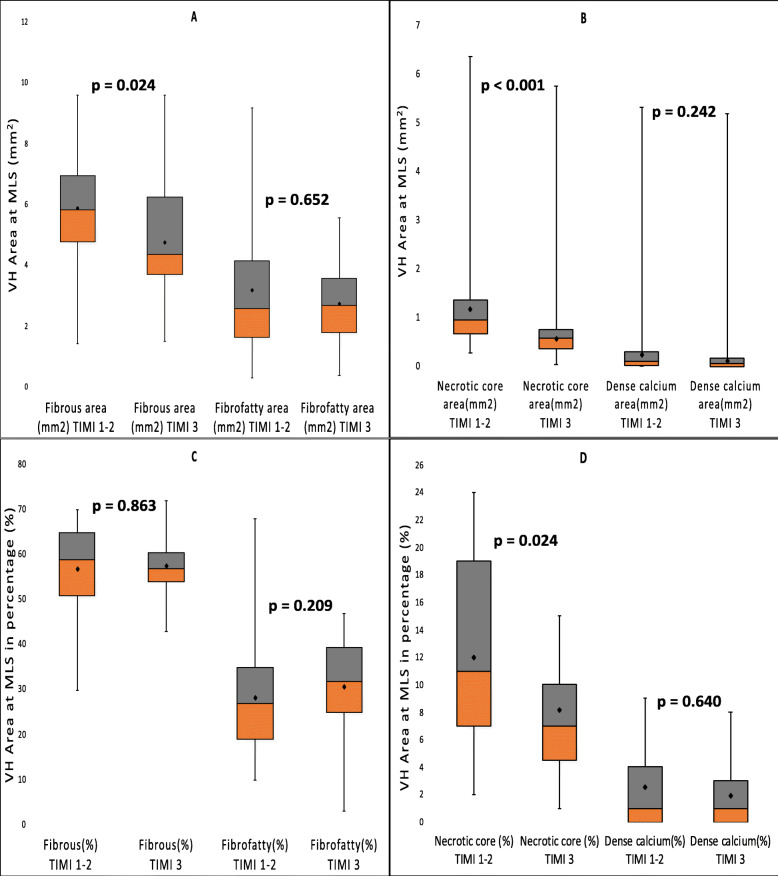
The virtual histology plaque components in TIMI 1–2 and TIMI 3 groups at the minimum lumen area site (MLS). **a**, **b** The absolute plaque components and **c**, **d** the relative plaque components at the MLS

**Fig. 2:**
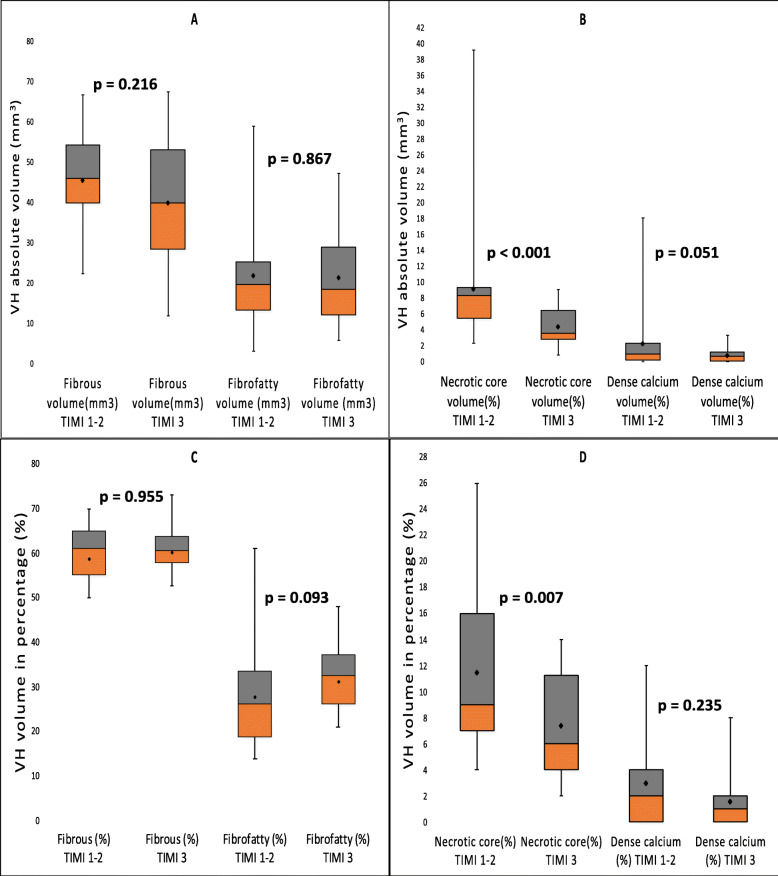
The virtual histology plaque components in TIMI 1–2 and TIMI 3 groups over the segment. **a**, **b** The absolute volumes of the plaque components and **c**, **d** the relative plaque components

**Fig. 3:**
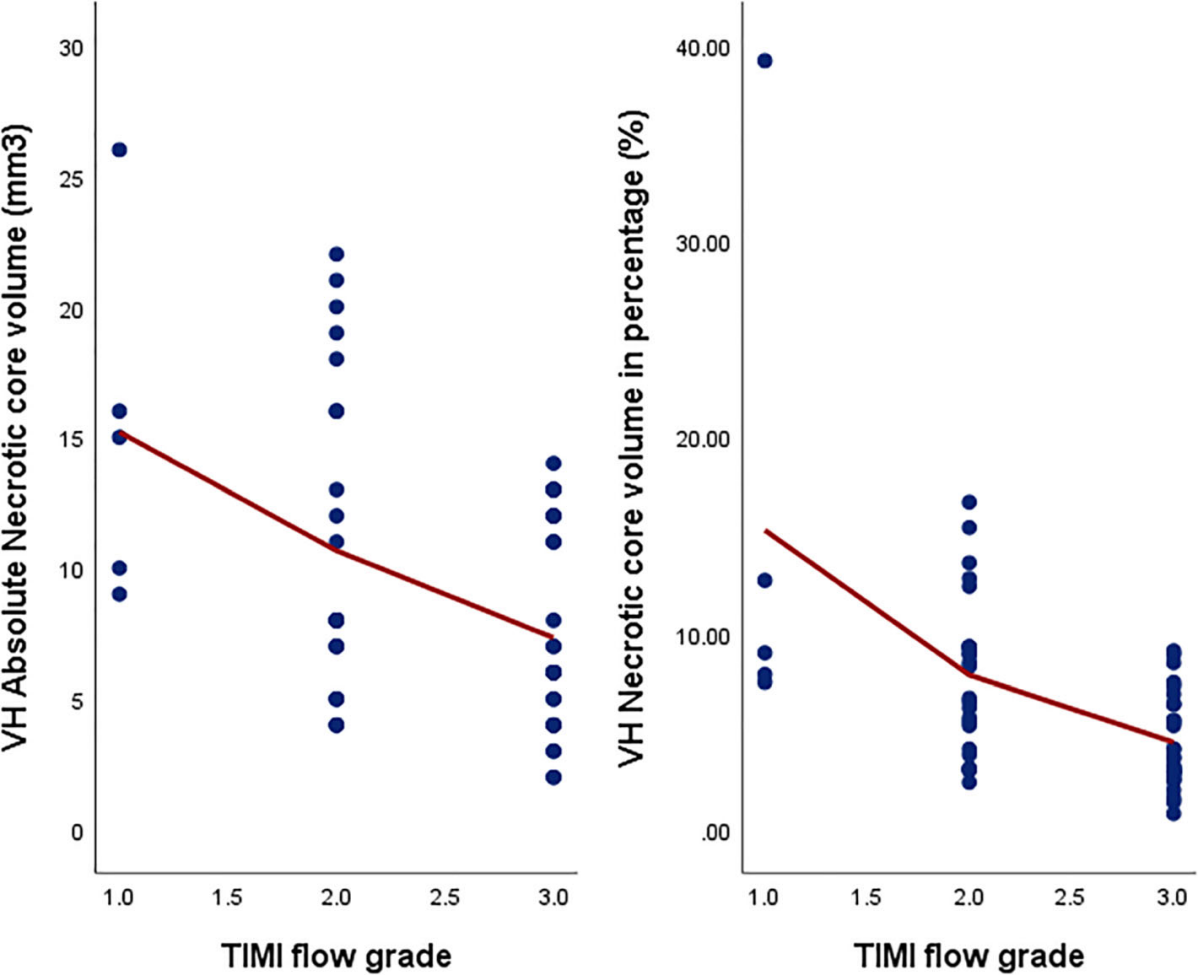
Absolute necrotic core volume (left panel) and relative necrotic core percentage (right panel) in the culprit plaque, according to the post-fibrinolysis TIMI flow grade

The VH-IVUS results are displayed in Figs. 1 and 2. At the minimal luminal area site (MLS), fibrous area, necrotic core area, and necrotic core percentage were notably higher in the TIMI 1–2 groups as compared to the TIMI 3 group (fibrous area: 5.83 mm^2^ vs. 4.37 mm^2^, *p* = 0.024; NC area: 0.95 mm^2^ vs. 0.59 mm^2^, *p* < 0.001, and NC percentage 11.0% vs. 7.1%, *p* = 0.024, respectively). Similarly, the absolute necrotic core volume and necrotic core percentage were significantly higher in the TIMI 1–2 groups in comparison to the TIMI 3 group (absolute NC volume: 8.3 mm^3^ vs. 3.65 mm^3^, *p* < 0.001; NC percentage: 9.3% vs. 6.0%, *p* = 0.007, respectively). Correlation of the absolute necrotic core volume and relative necrotic core percentage with the TIMI flow grades is depicted in Fig. 3. The occurrence of TCFA either single or multiple did not differ between the two groups (single TCFA: 29.0% vs. 20.7%, *p* = 0.462 and multiple TCFAs: 12.9% vs. 3.4%, *p* = 0.355, respectively).

### Predictors and determinants of TIMI flow grade

The variables with significant values in the bivariate analysis were subjected further for multivariate analysis. We performed backward stepwise logistic regression and the model summary statistics, Cox and Snell *R*^2^ was 0.303, and Nagelkerke *R*^2^ was 0.404. The prediction accuracy for this model was 72.2%, and the overall model was significant with a *p* < 0.001. The variables taken for multivariate analysis were distal reference plaque burden, lesion EEM CSA, fibrous area at MLS, necrotic core area at MLS, necrotic core percentage at MLS, and necrotic core volume. On multivariate analysis, the absolute NC volume was found to be the only independent predictor of TIMI 1–2 flow post fibrinolysis in STEMI patients (odds ratio = 1.561; 95% CI 1.202–2.026, *p* = 0.001).

**Fig. 4:**
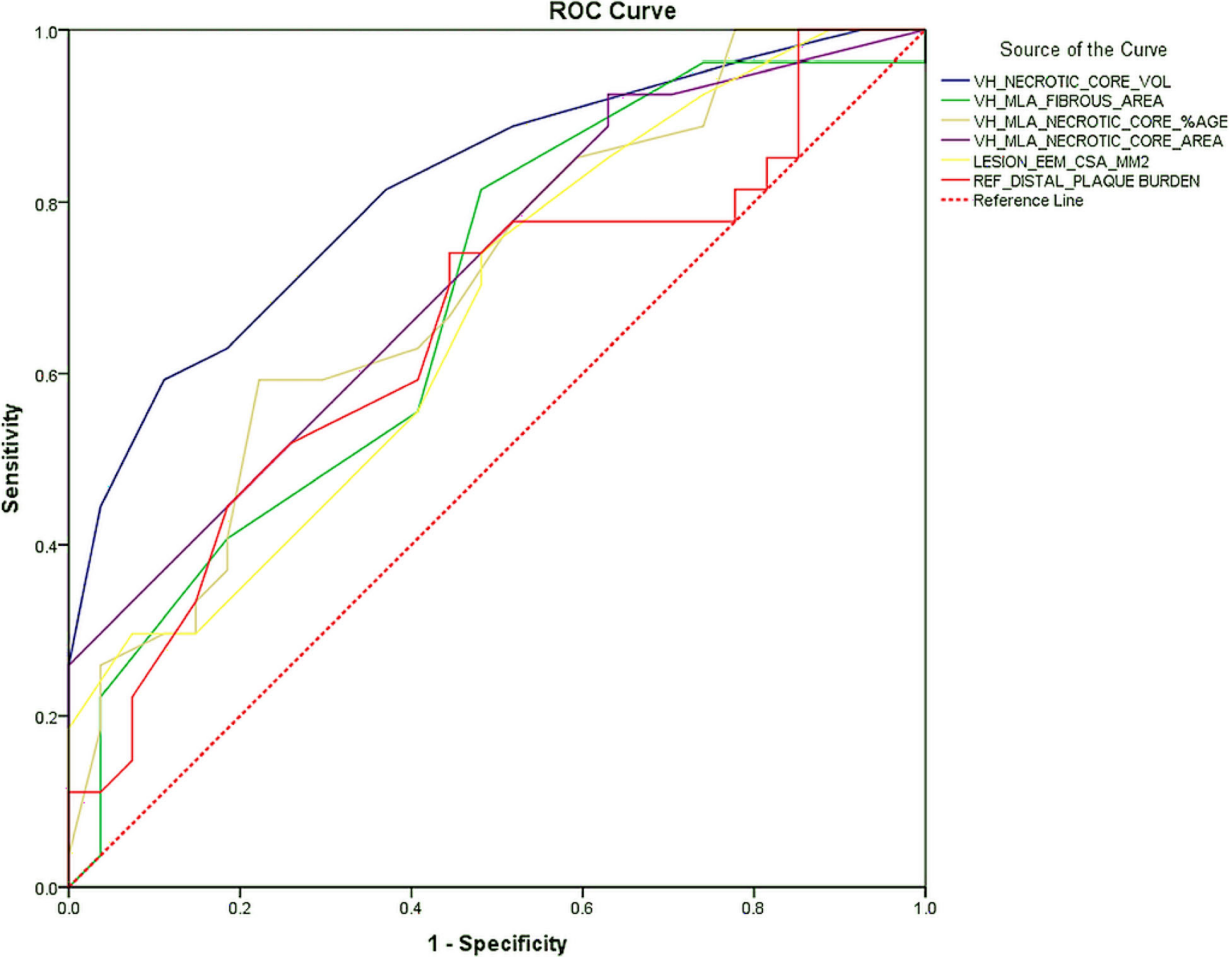
Receiver operating characteristic (ROC) curves of the gray-scale and VH-IVUS parameters for the determinants of TIMI 1–2 flow

Receiver operating characteristic (ROC) curve analyses were undertaken to single out the gray-scale IVUS (distal reference plaque burden, lesion EEM CSA) and VH-IVUS (fibrous area at MLS, necrotic core area at MLS, necrotic core percentage at MLS, and absolute necrotic core volume) parameters that could assist differentiating cases of TIMI 1–2 flow from TIMI 3 flow post fibrinolysis in STEMI (Fig. 4). The absolute necrotic core volume had the best predictive value (AUC = 0.816, *p* < 0.001) for TIMI 1–2 flow post fibrinolysis, and the best cutoff value to predict TIMI 1 or 2 flow was > 4.5 mm^3^ with a sensitivity and specificity of 79% and 61%, respectively.

## Discussion

The main findings of the current study are as follows: (i) the necrotic core component of plaque in culprit lesion in patients with STEMI after successful fibrinolysis is strongly associated with the extent of flow restoration in the culprit artery. (ii) The necrotic core volume was the only independent predictor of TIMI 1–2 flow post fibrinolysis in STEMI patients.

Atherosclerosis is a continuous process developing in arterial wall lesions with progressive accumulation of cholesterol-rich lipid deposits along with the inflammatory response [21]. The patients with ACS presents with a varied spectrum in terms of clinical presentations, intracoronary imaging, and pathological findings [22]. Autopsy series in patients with sudden cardiac death have shown the frequency of coronary thrombus in 60% with underlying etiology detected to be plaque rupture (50–60%), plaque erosion (30–35%), and calcified nodule (2–7%) [23]. Similarly in vivo studies in ACS and STEMI using various intracoronary imaging modalities have shown the incidence of plaque rupture varying from 44 to 73% and plaque erosion in 27–44% [22].

Plaque rupture tends to occur at the weak and thinnest portion of the fibrous cap with maximum infiltration of macrophage foam cells. The activated macrophages secrete proteolytic enzymes such as plasminogen activators, cathepsins, and matrix metalloproteinases (MMPs). Thinning of the fibrous cap involves a gradual loss of smooth muscle cells (SMCs) and degradation of collagen matrix by the infiltrating macrophages [24]. Intraplaque hemorrhage arises from vasa vasorum that infiltrates the plaque from the adventitia in response to the hypoxic environment and plays an important role in plaque vulnerability [25]. Further, it promotes inflammation and increases the level of free cholesterol leading to plaque progression and rapid necrotic core expansion leading to rupture [24, 26, 27]. The rupture of the fibrous cap exposes the lipids and tissue factors in the necrotic core to the thrombogenic factors of bloodstream [28].

Pathological studies with 2 to 3 mm interval sections revealed plaque ruptures to be frequently situated at a severely narrowed site or distal to it [24, 29, 30]. The site of plaque rupture in STEMI is usually not at the severe stenosis but is proximal to the narrowest portion. Plaque ruptures occur predominantly in the vicinity of the maximum necrotic core site and tend to be proximal to the maximum plaque burden and minimum lumen area sites. The ruptures occurred at the maximum necrotic core sites in 26% and either proximal (44%) or distal (30%) to it in the remaining 74% suggesting that the longitudinal shoulder of the maximum necrotic core site is the weakest point for plaque rupture [31]. Few other studies also have shown that the maximum necrotic core site is located proximal to the severe most stenosis [32, 33]. Therefore, the above data suggests that the analysis of the most diseased segment in and around the lesion provides useful information regarding the events linked to plaque rupture.

Autopsy series, in sudden coronary death patients revealed higher necrotic core content in culprit plaque and rupture-prone plaques [34]. The necrotic core content of the culprit lesion tends to be significantly higher in ACS as compared to stable angina and is considered as a marker of plaque vulnerability [35, 36]. Plaque rupture or erosion leads to the exposure of the necrotic core contents into the blood circulation causing activation of tissue factor and coagulation cascade, subsequently culminating into coronary thrombosis and ACS. Further, the embolization of the gruel necrotic core components along with thrombi distally leads to clogging of microcirculation potentiating coronary slow flow [37, 38].

On the gray-scale IVUS analysis, a larger plaque burden has been identified as a predictor and discriminator of coronary slow flow in ACS undergoing PCI [39, 40]. However, in our study, the plaque burden was comparable between both groups. Coronary artery remodeling is a vascular responsive phenomenon varying from positive remodeling frequently observed in ACS to negative remodeling associated with stable angina [41, 42]. Positive remodeling, a marker of plaque vulnerability, with its high lipid content and macrophage count is a potential risk factor for slow flow after primary PCI and subsequent cardiac enzyme elevation [43, 44]. In our study, positive remodeling was observed in both groups with no difference.

On virtual histology, the necrotic core comprises cholesterol crystals, lipid-laden foam cells, microcalcifications, and microhemorrhages. The substantial increase of these elements noted in STEMI with plaque rupture when embolized to distal coronary microcirculation contributes to slow flow [13, 14, 45]. Giannopoulos et al. showed that the relative necrotic core percentage by VH-IVUS in culprit lesions with STEMI are linked to coronary flow restoration following thrombolysis and was significantly higher in patients with TIMI flow grades 1–2 [46]. In line with the above findings, the present study also showed a higher relative necrotic core percentage. Additionally, we also demonstrated that the absolute necrotic core volume to be higher in the TIMI 1–2 groups, a finding not observed previously [46]. Souza et al. on tissue characterization by iMAP in culprit lesions with STEMI after fibrinolysis revealed a predominance of necrotic core component demonstrating greater plaque vulnerability and instability [47]. Studies involving optical coherence tomography (OCT) in individuals presenting with ACS and STEMI have identified lipid-rich plaque content as an important risk factor for coronary slow flow after stent deployment and also a predictor of blood flow restoration after fibrinolysis for STEMI [38, 40, 48].

Coronary calcification in general indicates a long-standing atherosclerotic disease and its extent correlates with atherosclerotic plaque burden [49, 50]. The inflammatory mediators and lipid content within atherosclerotic plaque induce osteogenic differentiation of vascular smooth muscle cells resulting in atherosclerotic intimal calcification in the vessel wall [51, 52]. The effect of calcification on future coronary events appears to be biphasic with spotty and superficial calcification being more vulnerable for plaque rupture leading to acute coronary syndrome. On the contrary, large calcium deposits were seen more frequently in stable angina pectoris suggesting that as the calcified plaques coalesce, the interface area decrease, and mechanical stability of the plaque increases [49, 52–55]. Calcification also contributes to the slow flow phenomenon after plaque rupture by distal embolization. However, previous studies have shown that dense calcium on VH-IVUS did not contribute to coronary slow flow post-fibrinolysis [46, 47]. Although in our study the dense calcium was high in the TIMI 1–2 groups, it failed to reach the statistical significance and this further substantiates the existing literature.

### Study limitations

Firstly, the study was a single-center prospective observational study with the inclusion of a relatively small sample size. However, the study achieved a statistical significance to demonstrate the difference in the plaque phenotypes. Secondly, an important drawback of VH-IVUS is the inherent and inappropriate classification of thrombus as fibrous/fibrofatty phenotype. The effect of this was minimal as no difference was evident in either the fibrous or fibrofatty component over the analyzed segment among the studied groups. Further presence of thrombus underestimates the incidence of TCFAs. A long-term clinical follow-up for outcomes is warranted in these subsets.

## Conclusion

The necrotic core content of the plaque in culprit lesions in patients with STEMI as assessed by VH-IVUS determines the adequacy of flow restoration after fibrinolysis. Our study demonstrates that absolute necrotic core volume was the only independent predictor of flow restoration following fibrinolysis in STEMI. The identification of increased necrotic volume as demonstrated helps in the management and prognosis of patients who might end up with less than normal coronary flow or slow flow.

## Data Availability

All data used and analyzed in the current study were available in the institute. These data are available from the corresponding author on reasonable request.

## Abbreviations

ACS: Acute coronary syndrome
CVD: Cardiovascular disease
CK-MB: Creatine kinase-myocardial band
CTFC: Corrected thrombolysis in myocardial infarction frame count
CSA: Cross-sectional area
DC: Dense calcium
EEM: External elastic membrane
EEM CSA: External elastic membrane cross-sectional area
IVUS: Intravascular ultrasound
MMPs: Matrix metalloproteinases
MLS: Minimal luminal area site
NC: Necrotic core
NSTEMI: Non-ST-segment elevation myocardial infarction
OCT: Optical coherence tomography
P&M CSA: Plaque and media cross-sectional area
PPCI: Primary percutaneous coronary intervention
QCA: Quantitative coronary angiography
ROC: Receiver operating characteristic
SMCs: Smooth muscle cells
STEMI: ST-segment elevation myocardial infarction
TIMI: Thrombolysis in myocardial infarction
TCFA: Thin-cap fibroatheroma
VH-IVUS: Virtual histology intravascular ultrasound
