# Correction to: Association of culprit lesion plaque characteristics with flow restoration post-fibrinolysis in ST-segment elevation myocardial infarction: an intravascular ultrasound-virtual histology study

**DOI:** 10.1186/s43044-020-00125-6

**Published:** 2020-12-20

**Authors:** Raghavendra Rao K, Sreenivas Reddy, Jeet Ram Kashyap, Vadivelu Ramalingam, Debabrata Dash, Vikas Kadiyala, Suraj Kumar, Hithesh Reddy, Jaspreet Kaur, Ashok Kumar, Naindeep Kaur, Anish Gupta

**Affiliations:** 1 Department of Cardiology, Government Medical College and Hospital, Sector 32, Chandigarh, 160030 India; 2 Department of Neurology, Post Graduate Institute of Medical Education and Research (PGIMER), Chandigarh, 160012 India

**Correction to: Egypt Heart J 72, 86 (2020)**

**https://doi.org/10.1186/s43044-020-00121-w**

Following publication of the original article [1], the authors identified an error in the labelling of Figs. 1 and 2. The corrected versions of these figures have been published in this correction.

**Fig. 1:**
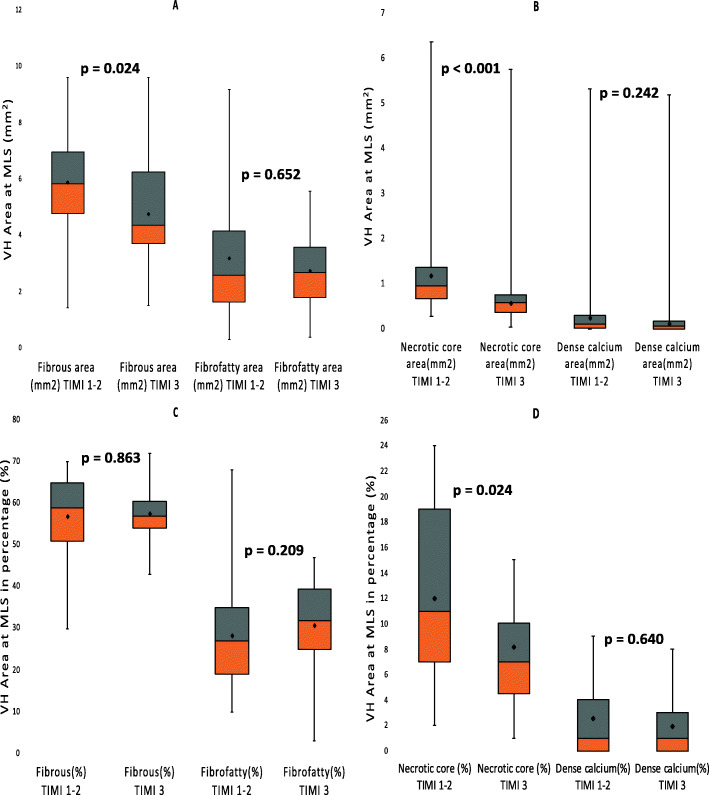
The virtual histology plaque components in TIMI 1–2 and TIMI 3 groups at the minimum lumen area site (MLS). **a** and **b** represents the absolute plaque components and **c** and **d** represents the relative plaque components at the MLS

**Fig. 2:**
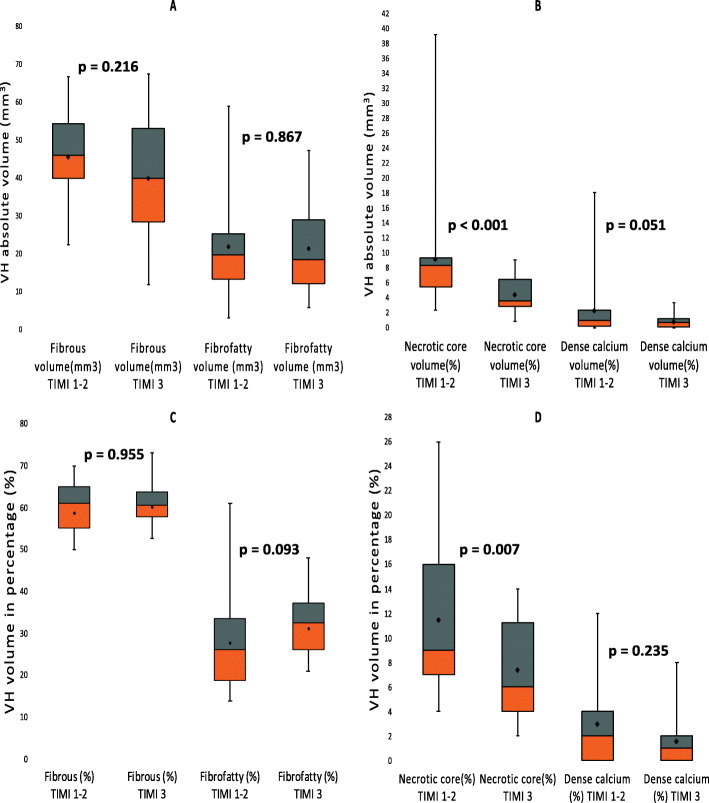
The virtual histology plaque components in TIMI 1–2 and TIMI 3 groups over the segment. **a** and **b** represents the absolute volumes of the plaque components and **c** and **d** represents the relative plaque components

The original article [1] has been corrected.
